# Intra-and-Inter Species Biomass Prediction in a Plantation Forest: Testing the Utility of High Spatial Resolution Spaceborne Multispectral RapidEye Sensor and Advanced Machine Learning Algorithms

**DOI:** 10.3390/s140815348

**Published:** 2014-08-20

**Authors:** Timothy Dube, Onisimo Mutanga, Elhadi Adam, Riyad Ismail

**Affiliations:** 1 Discipline of Geography, School of Agricultural, Earth and Environmental Sciences, University of KwaZulu-Natal, P/Bag X01, Scottsville, Pietermaritzburg 3209, South Africa; E-Mails: MutangaO@ukzn.ac.za (O.M.); Riyad.Ismail@sappi.com (R.I.); 2 University of Witwatersrand, Geography and Environmental Studies Division School of Geography, Archaeology and Environmental Studies, Room 102-Bernard Price Building, East Campus, Braamfontein Johannesburg 2050, South Africa; E-Mail: Elhadi.Adam@wits.ac.za

**Keywords:** bag fraction, biosphere-atmospheric interactions, learning rate, high resolution RapidEye imagery, tree complexity, variable importance and variable selection

## Abstract

The quantification of aboveground biomass using remote sensing is critical for better understanding the role of forests in carbon sequestration and for informed sustainable management. Although remote sensing techniques have been proven useful in assessing forest biomass in general, more is required to investigate their capabilities in predicting intra-and-inter species biomass which are mainly characterised by non-linear relationships. In this study, we tested two machine learning algorithms, Stochastic Gradient Boosting (SGB) and Random Forest (RF) regression trees to predict intra-and-inter species biomass using high resolution RapidEye reflectance bands as well as the derived vegetation indices in a commercial plantation. The results showed that the SGB algorithm yielded the best performance for intra-and-inter species biomass prediction; using all the predictor variables as well as based on the most important selected variables. For example using the most important variables the algorithm produced an R^2^ of 0.80 and RMSE of 16.93 t·ha^−1^ for *E. grandis*; R^2^ of 0.79, RMSE of 17.27 t·ha^−1^ for *P. taeda* and R^2^ of 0.61, RMSE of 43.39 t·ha^−1^ for the combined species data sets. Comparatively, RF yielded plausible results only for *E. dunii* (R^2^ of 0.79; RMSE of 7.18 t·ha^−1^). We demonstrated that although the two statistical methods were able to predict biomass accurately, RF produced weaker results as compared to SGB when applied to combined species dataset. The result underscores the relevance of stochastic models in predicting biomass drawn from different species and genera using the new generation high resolution RapidEye sensor with strategically positioned bands.

## 1. Introduction

Forests serve as an important key driver of regional and local climate systems through biosphere-atmospheric interactions [1–3]. Information on forest spatial distribution, biomass levels and dynamics is therefore, required for accurate estimation of greenhouse gases flux, policy development and implementation [4]. In addition, knowledge on intra-and-inter commercial forest biomass is central in: (i) determining their productive capacity; (ii) ensuring informed sustainable management practices and (iii) understanding the functioning of the planet and the environment [5,6]. Therefore, continuous estimation, mapping and monitoring of forest aboveground biomass (tonnes ha^−1^), which is the amount of living plant matter [5]; is central in climate modelling worldwide, due to its significance in net carbon emission computations [7–9].

Currently, there are two approaches for forest biomass estimation namely, field-based traditional methods (*i.e.*, field measurements or harvesting) and remotely sensed methods [10]. So far, traditional methods have been side lined in favour of remotely sensed techniques; since its inception. Although, regarded as highly accurate [10,11]; the traditional methods are exceedingly time consuming, labour intensive, and difficult to implement, especially in remote areas and are practically and spatially limited to a small tree sample size and requires a sufficient number of samples [10,12]. Recent evidence suggests that remote sensing seems to be a valuable and low-cost tool for determining forest biophysical attributes when compared to field surveys [13–15]. Remotely sensed data permits robust biomass retrieval which is critical for assessing the ecosystem yield and carbon accounting. As a result, biomass estimation using remotely sensed data as the primary source has gained increasing interest in the past decades especially for natural forests at both local and regional scales [10].

Although biomass cannot be directly quantified from space, satellite image reflectance permits the extraction of biomass estimates especially when integrated with field-based measurements [15]. Consequently, various remotely sensed studies concerning forest biomass estimation have been applied at different scales. It has been discovered that coarse spatial resolution optical sensors are useful for biomass mapping at continental and global scale rather than at local scale [16,17] due to the plausible trade-off between spatial resolution, image coverage and frequency in data acquisition [3,6,10,18,19]. The main limitation with the broadband multispectral sensors for biomass estimation is the fact that they are characterised by mixed pixels which occur as a result of large sensor footprint [20,21]. In that regard, the huge difference between the satellite data pixel size and the ground reference data makes these sensors inapplicable for intra-and-inter species biomass prediction in commercial forest plantations.

Recent efforts have been geared towards the use of high resolution sensors such as narrow band hyperspectral, radar and lidar data for estimating aboveground forest biomass (AGB) to reduce the limitations associated with the broadband multispectral data sets [21–27]. Results have shown that hyperspectral, radar and lidar sensors have robust means of data collection and subsequent characterization of the vertically distributed forest attributes hence can be regarded as an appropriate primary data source for forest inventorying. The use of these data sets nonetheless comes with its own limitations in terms of cost; availability; spectral contiguity, processing and analysis complexity especially in the African context given its economic situation and lack of the required technical expertise. For example, processing hyperspectral data for vegetation applications is a major challenge due to the Hughes phenomenon or “the curse of dimensionality”. This problem often introduces a high degree of multicollinearity as a result of the similarities in the biophysical spectral reflectance properties [28–31].

The utility of new generation sensors, such as the RapidEye with strategic bands is therefore seen as a trade-off between the advantages of coarse multispectral data, hyperspectral, lidar and radar data in predicting intra-and-inter species AGB [32–34]. Currently, RapidEye together with WorldView-2 sensors are the only commercial multispectral satellite sensors which provide a reasonable number of spectral bands that are configured in unique portions of the electromagnetic spectrum and provide a global, high-resolution access to the red- edge spectral band [35]. In remote sensing, the “red-edge” is the region of abrupt change in the leaf reflectance between 680 and 780 nm, due to the combined effects of strong chlorophyll absorption in red wavelengths and high reflectance in the NIR wavelengths due to leaf internal scattering [36]. The new generation RapidEye image containing strategically positioned bands with a fine spatial resolution of 5 m is hypothesized to be critical for vegetation mapping when compared to the traditional broadband satellite images, such as ASTER, SPOT and Landsat Thematic Mapper. Above all, the RapidEye reduces unnecessary redundancy, a problem associated with hyperspectral data [13]. Recently, the strategically positioned bands of the RapidEye imagery has successfully been applied extensively in detecting different levels of insect defoliation in Mopane woodlands [37,38] whereas other studies have demonstrated that the strategically positioned RapidEye bands allow for enhanced vegetation mapping [39,40].

However, the rich spectral information contained in this data set has not been exploited for estimating intra-and-inter species biomass in managed commercial plantations. For instance, commercial forests with mixed species (inter-species) are characterised by significant biomass variations, making it difficult for national carbon accounting. Taxonomical and structural differences are a major problem for intra-species aboveground biomass estimation [21]. More importantly, different species and genera result in high biomass variations that are associated with non-linear relationships making algorithm applications a significant challenge in estimating ABG in such environments. Due to the intra-and-inter species variability there is a high probability of outliers and unbalanced data sets in the collected training data. It is therefore critical for biomass studies to identify robust models that could overcome the failure to estimate biomass in forests characterised with intra-and-inter species [21,41–43].

In this study we therefore assessed the potential of two machine learning algorithms; Stochastic Gradient Boosting (SGB) and Random Forest (RF) in predicting intra-and-inter species biomass in a commercial plantation forest in the midlands region of KwaZulu-Natal, South Africa using the strategically positioned spectral information derived from 5 m RapidEye imagery. Previous studies have shown that non-parametric statistical techniques such as the SGB and RF simplify the biomass estimation process when compared to other statistical regression methods [12,13,21]. Both regression ensembles have received considerable attention due to a number of statistical modeling properties. For instance, the SGB method produces results with plausible and highly robust estimates in regression studies due to its ability to handle outliers, inaccurate training data, missing and unbalanced data sets [44–46]. Moreover, the model's stochastic characteristic in modelling non-linear relationships and the inherent ability to handle, identify as well as select critical variables from large amounts of data is expected to provide the best model accuracies [21,45–47]. Most importantly, SGB uses a stage-wise additive model fitting procedure that enhances the predictive performance of weak learning algorithms.

On the other hand, RF provides other appealing statistical properties, such as the useful internal estimates of error, strength, correlation and variable importance [48,49]. In addition, Strobl, Boulesteix, Kneib, Augustin and Zeileis [49] describe random forest algorithm as an effective tool which performs simple and complex regressions with modest fine-tuning of parameters resulting in accurate predictions. The highlighted characteristics of SGB and RF as well as the probability of intra-and-inter species biomass variability have therefore prompted an investigation of their capabilities (SGB and RF) in predicting AGB from a commercial forest in the midlands of KwaZulu Natal, South Africa. Although both machine learning techniques have been found to be robust under certain conditions, in this mixed species environment of KwaZulu Natal, it is expected that SGB would perform better, due to its capabilities in modelling possible outliers and unbalanced data sets as well as non-linear relationships. To the best of our knowledge, so far no study has assessed the SGB and RF for intra-and-inter species biomass prediction in a commercial forest and in particular, using the strategically positioned bands of the new generation sensors such as RapidEye. Therefore, our main objective was to investigate the robustness of the two machine learning algorithms in predicting intra-and-inter species biomass from a plantation forests using the recent high spatial resolution spaceborne RapidEye multispectral imagery. A secondary objective was to evaluate the relative importance of the high resolution RapidEye reflectance bands as well as the derived vegetation indices in the prediction of intra-and-inter species biomass.

## 2. Materials and Methods

### 2.1. Study Area

**Figure 1.**
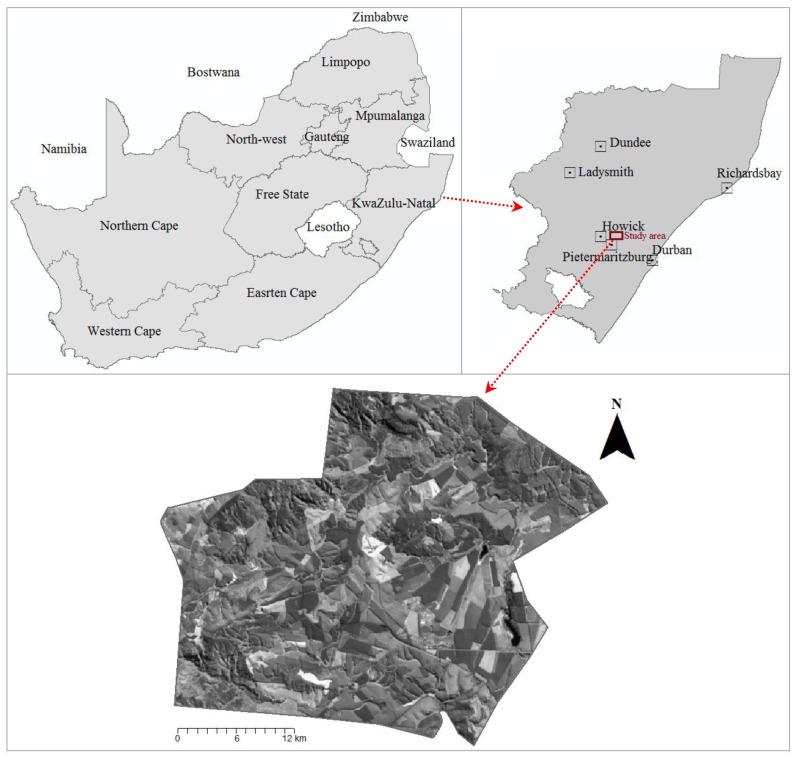
Location of Sappi Clan forest in the midlands of KwaZulu Natal, South Africa.

The study was conducted at sappi Clan forest, located approximately 27 km away from Pietermaritzburg city, the provincial capital of KwaZulu-Natal Province, South Africa (Figure 1). The plantation is located between Latitudes (29°24′46.74″S, 29°17′45.94″S) and Longitude (30°18′32.89″E, 30°28′28.21″E). South Africa is home to vast tracks of commercial plantation forests, both hardwood and softwoods, covering approximately one percent of the total land area [50]. Specifically, the Clan forest used in this study covers about 6700 ha. The forest is characterized by extensive commercial forestry dominated by *Pinus* (*P. taeda*), and *Eucalyptus spp.* (e.g., *E. grandis* and *E. dunii*). The climate in the study area is sub-tropical with the mean annual rainfall varying between 700 mm and 1500 mm [51]. These fast-growing *Eucalyptus* species are planted with clones or seedlings and harvested every six to seven years. Stands are managed on a pulpwood regime (*i.e.*, established at 1667 trees/ha); and intensive soil preparation and weed control measures are practiced, until crown closure occurs between 1 to 1.5 years.

### 2.2. Field Data Collection and Sampling Design

**Figure 2.**
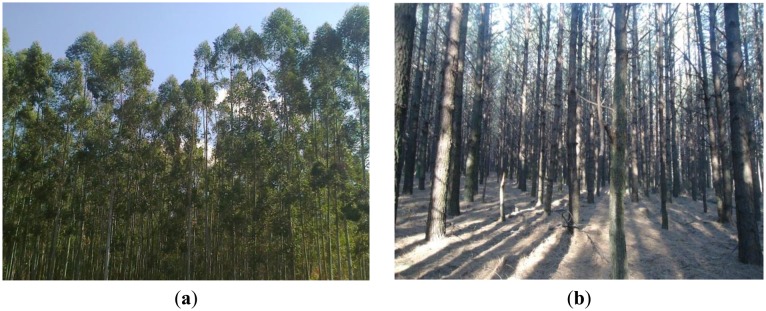
Typical field site showing (**a**) *Eucalyptus spp.* and (**b**), *P. taeda* in early August 2013.

The field campaign was carried out between the 30th of July and the 22nd of August 2013, in conjunction with Sappi annual routine-field surveys. Sampling was conducted on *Eucalyptus grandis, Eucalyptus dunii* and *Pinus taeda* forests aged between 8 and 20 years (Figure 2a,b). Selected tree structural variables, namely; tree diameter-at-breast height (DBH), tree height (H) were measured for each plot (181) using the Haglof Digitech Calliper and Vertex IV laser instrument respectively. A total of 181 plots were selected for field surveys using vector maps, courtesy of Sappi. The selection criteria were based on species type, age and spatial location of compartments. The measurements were collected using a grid-based systematic sampling technique, utilizing a circular sample plots, approximately 400 m^2^ in size. These plots were systematically distributed (usually every 100 m) within the stand. Sample intensities varied between 2% and 10%, depending on the species composition, stand size or local forest conditions [52].

### 2.3. Field Aboveground Forest Biomass Computation

For individual species biomass (t·ha^−1^) calculation, two approaches were applied for the three selected species (*E. grandis, E. dunii* and *P. taeda*). The first method was only used for *Eucalyptus spp.* and it involved the use of volume and biomass expansion factors found in literature specifically for South African species [53]. Volume was derived and reported at stand level following the allometric method explained by Bredenkamp [54]. For *P. taeda*, a general allometric equation was used for biomass computation, as proposed by the Intergovernmental Panel on Climate Change (IPCC), IPCC [55]. The basis for the application of this allometric equation for this species *(P. taeda)* in particular is the fact that the rainfall (800–1500 mm) and temperature range (21 °C–34 °C) are similar to the climatic conditions prevailing in the study area. The equation used for the species was also formulated using diameter-at-breast height (DBH) ranging from 0.6 cm–56 cm at rainfall of 800 to 1500 mm and temperatures were similar to the study area. Species difference prompted the use of different approaches for computing biomass because of the existing differences in species structural and taxonomical characteristics [11,41,43]. Moreover, literature shows that different allometric equations exist for biomass computation for the selected species [11,56]. The biomass results from the two approaches were finally standardised to the same unit of measurement, which is tonnes per hectare (t·ha^−1^).

### 2.4. Image Acquisition and Data Preprocessing

A recent high spatial resolution spaceborne multispectral sensor (*i.e.*, RapidEye imagery) with zero percent cloud cover, covering the study area was obtained on the 25th of August 2013 from DLR Germany. The RapidEye image comprised of five multispectral bands with a spatial resolution of 5 m. The spectral ranges of the five bands are 440–510 nm (B1-blue), 520–590 nm (B2-green), 630–685 nm (B3-red), 690–730 nm (B4-red-edge), and 760–850 nm (B5-near infrared). All the RapidEye products are collected by a 12 bit imager. Radiometric corrections were applied to the RapidEye image, subsequently converting the image digital numbers (DN) into values directly related to absolute radiances, using a constant factor (originally determined during launch) [57]. Earlier experimentation done by Naughton, *et al.* [58] demonstrated that the image registration error was within a single pixel, hence further geometric processing was not implemented. Radiance image was atmospherically corrected and transformed to canopy reflectance using the Fast Line-of-Sight Atmospheric Analysis of Spectral Hypercubes (FLAASH) algorithm built in ENVI 4.7 software [59].

### 2.5. Spectral Information and Vegetation Indices Derived from Strategically Positioned Multispectral Spaceborne RapidEye Image Bands

A point map of the biomass plots was developed using the field data and GPS recordings. This map was then overlaid on the RapidEye images to generate a region-of-interest (ROI) map using the central GPS point for each plot (*n* = 181). A 3 × 3 pixels window (*i.e.*, 15 m × 15 m) was used to collect vegetation image spectra from each band (*n* = 5) using ArcGIS 10.2 software. The 3 × 3 pixels window size was used in order to avoid the inclusion of pixels located outside the plot [13,60]. Hence, only pixels that fall entirely within the ROIs were included in the spectral dataset, while the pixels that partially fall inside the ROIs were discarded [13,60,61]. The spectra were collected and averaged for each plot. All derived parameters that were related to the field plot data are listed in Table 1. The indices were chosen, based on previous research dealing with forest biomass estimation from remote sensing data.

### 2.6. Intra-and-Inter Species Biomass Training and Test Datasets

To validate the performance of the SGB and RF algorithms the datasets (*E. dunii*: *n* = 63, *E. grandis*: *n* = 65, *P. taeda*: *n* = 53 and all species: *n* = 181) were randomly split into 70%, training dataset and 30% for a test (independent) dataset [12,62]. Moreover, the training datasets were used in optimizing both regression algorithms (SGB and RF) and to train the prediction models whereas the test dataset was used to examine the performance and reliability of the prediction model.

### 2.7. Statistical Analysis

Two main data analysis techniques were implemented and these include stochastic gradient boosting (SGB) and random forest (RF) regression algorithms. The two algorithms are discussed in detail below.

#### 2.7.1. Stochastic Gradient Boosting Regression Model

Stochastic gradient boosting is a powerful machine learning technique producing competitive, highly robust and interpretable procedures for both regression and classification applications [63]. The tree ensemble has the ability to accommodate different types of explanatory variables and data with missing values [45]. The ensemble is immune to outlier effects; it can fit complex nonlinear relationships and automatically handles interaction effects among predictors. The algorithm introduces also an element of stochasticity, thus improving model accuracy and reducing over-fitting [44,47].

SGB predicts the response variables by combining regression tree and boosting algorithms [44,45,47,73,74]. The ensemble uses a backward stage-wise approach by fitting regression tree models iteratively to a subset of the training data (50%) that is randomly selected without replacement. A residual deviance is then calculated on data not used in the model fitting process. Trees are added until the total residual deviance calculated from the withheld data ceases to decrease. The number of trees giving the lowest total residual deviance represents the most appropriate model for prediction.

During model fitting SGB is governed by three important user-defined parameters [44,47,75] namely: (i) the learning rate (*lr*), which determines the contribution of each tree to the final model; (ii) the tree complexity (*tc*), which is the number of independent variables interacting to determine each split and (iii) the number of regression trees (*nt)* in the ensemble. The learning rate controls the increase in model complexity, with smaller values resulting in fitting a larger number of trees [45]. For each combination of *nt*, *tc* and *lr*, the combination producing the lowest cross-validated deviance is then identified, using the training dataset. For this study, we fitted SGB models, with varying values for *nt* (100–10,000), *lr* (0·1–0·0001), *tc* of 1 and 5, a bag fraction of 0.2–0.75 and evaluated the results across all categories of species biomass. The gbm library [73] for the R statistical package for statistical analysis [76] was utilized to implement SGB.

#### 2.7.2. Stochastic Gradient Boosting and Relative Variable Importance

For the accurate and simple prediction of inter-and-intra species biomass, the relative individual variable influence was determined to identify the smallest number of input variables (*p* = 19) that yielded the best predictive performance. This information is important because not all model input variables are equally relevant in the modelling process. In this regard, it is often suitable to learn or determine the relative influence of each input variable in predicting inter-and-intra species biomass. Based on SGB, the relative influence of model terms was calculated by the contribution of each variable in reducing overall model deviance [45,47]. Subsequently, variable selection was achieved by implementing a backward feature elimination approach to determine the most important spectral bands and vegetation indices required for accurate biomass prediction. More precisely, the approach develops a model which utilizes all the input predictor variables and then progressively eliminates input predictor variables with least relative influence. Additionally, all SGB models are optimized in terms of their *lr*, *tc* and *nt* hyper-parameters. The SGB model for predicting inter-and-intra species biomass was initially run using nineteen variables.

#### 2.7.3. Random Forest Regression Algorithms

Random forest (RF) is a machine learning technique developed by Breiman [77] that employs bootstrap aggregation, where a number of trees (*ntree*) are constructed based on a random subset of samples derived from the training data. RF regression algorithm utilizes bootstrap samples from the training data without pruning to grow a large number of decision trees [48,78,79]. These trees assign each variable (RapidEye band reflectance or vegetation index) to a response value (biomass), using the averaged estimates that the value receives from the collection of all trees [48]. The algorithm has an additional modification of selecting only a random subset of candidate features (*mtry*) to determine the split at each node of a tree. This ensemble method uses recursive partitioning, to create multiple regression trees (*ntree*) and then averages the results of all trees [77]. RF algorithm is easy to implement as only two parameters (*ntree* and *mtry*) need to be optimized based on the lowest root mean square error (RMSE) of prediction [77,80,81,82]. The *ntree* parameter, the number of regression trees grown based on a bootstrap sample of the observations (the default value is 500 trees) and *mtry* is the number of different predictors tested at each node (the default value is 1/3 of the total number of the variables). Thus, in this study the *ntree* parameter values were tested in increments of 500 to 2500 with a 500 interval whereas the *mtry* was tested in increments of 1 to 19.

Approximately one-third of the data which is not included in the bootstrapped training sample, called the out-of-bag (OOB) samples is then used to evaluate the RF model. A number of researchers have shown that the OOB samples offer unbiased estimates of the training error [12,37,48,77,82,83]. The permutation based variable importance follows the rationale that the random permutation of a predictor variable represents the absence of the variable from the model. Hence, the difference in prediction accuracy prior and after permuting a variable is used as a measure of importance. The number of observations predicted correctly, decreases substantially if the permuted variable is strongly associated with the response values. Grömping [84] provided a more detailed account of the random forest's variable importance measures, both from the theoretical understanding and from the perspective of computational advantages. The ensemble was implemented using the randomForest package [85] within the R statistical package version R-3.0.2 [76].

#### 2.7.4. Variables Selection Using Random Forest

Random forest measure the importance of each predictive variable using the mean decrease in accuracy that is calculated using the OOB sample data. However, the challenge was to select the fewest number of predictors that offer the best predictive power and help in the interpretation of the final model. In this regard, a backward feature elimination method (BFE) integrated with random forest regression as part of the evaluation process was implemented (RF) based on 1000 model runs. The BFE uses the ranking to identify the sequence in which to discard the least important predictors from the input data sets. The method starts with the entire variables (*p* = 19) and then progressively eliminates the least promising variable from the list. For each iteration, the model is optimized by selecting the best *mtry* and *ntree*, the least promising variable is eliminated and root mean square error is calculated. The smallest subset of variables with lowest RMSE is then selected to predict inter-species biomass. A comprehensive analysis of the predictive performance of different subsets of extracted RapidEye reflectance and vegetation indices was implemented to explore the role of the new generation sensor in predicting interspecies biomass as well as to test if the variables selection method implemented in this study can enhance the predictive performance of random forest regression model.

#### 2.7.5. Effectiveness of SGB and RF in Predicting Intra-and-Inter Species Biomass

To assess the effectiveness of SGB and RF algorithms in predicting either intra or inter species biomass in a commercial forest environment, the r-square (R^2^) and the root mean square error (RSME) were computed (Equation (1). A one-to-one relationship between measured and predicted AGB values was fitted with coefficients of determination (R^2^), and root mean square error (RMSE) values reported:

(1) $$\text{RMSE}=\sqrt{\frac{\sum_{i=1}^{n}{\left(X_{\text{measured}}-X_{\text{predicted}}\right)}^{2}}{n}}$$

where *X_measured_* is measured biomass values, *X_predicted_* is predicted biomass values and *i* represent each of the predictor variables included in the summation process (*p* = 19).

## 3. Results

### 3.1. Intra-and-Inter-Species Aboveground Biomass (t•ha^−1^)

Table 2 shows descriptive statistics for each category of the target species (e.g., *E. dunii* (*n* = 63), *E. grandis* (*n* = 65), *P. taeda* (*n* = 53) and for the all species-datasets (*n* = 181). High biomass was observed for *P. taeda*, followed by *E. grandis* and *E. dunii* having the least biomass.

### 3.2. Intra-Species AGB: SGB and RF Regression Predictive Performance Based all Variables

**Figure 3.**
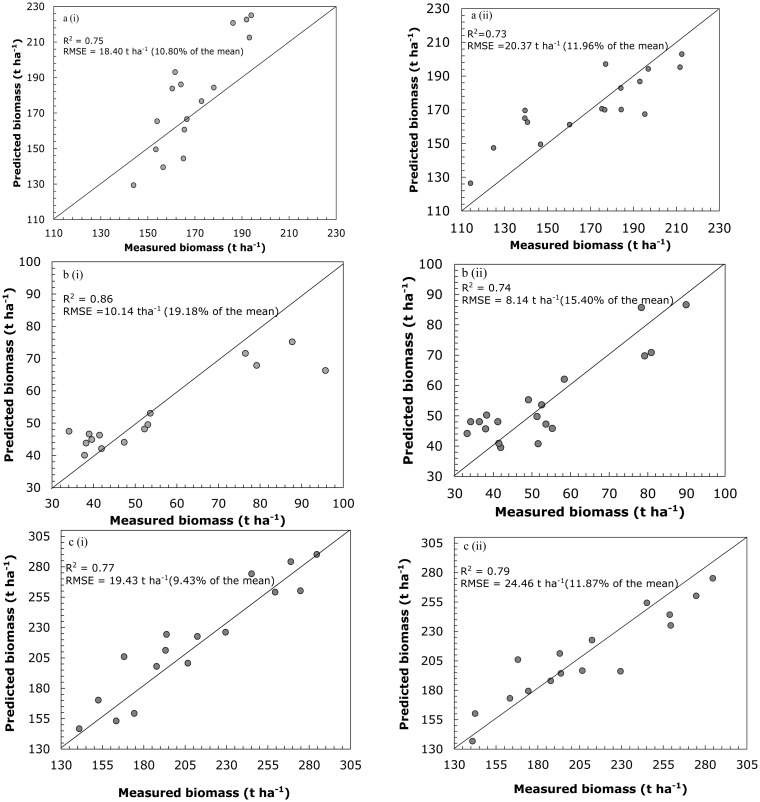
One-to-one relationship between measured and predicted intra-species biomass based on (i) SGB and (ii) RF algorithms. a, b, and c represent *E. grandis*, *E. dunii*, and *P. taeda* based on all the predictor variables (*p* = 19).

One to one relationship between measured and predicted intraspecies biomass using SGB and RF regression models are shown in Figure 3. For each model, the R^2^ and RMSE were reported. A comparative analysis of the predictive performance of the two models shows that the SGB model yielded better predictions for the intra-species dataset, producing R^2^ of 0.75 and RMSE of 18.40 t·ha^−1^ (10.80%) for E. grandis; R^2^ of 0.77 and RMSE of 19.43 t·ha^−1^ (19.18%) for *P. taeda*. Comparatively, the RF produced better results for *E. dunii* (R^2^ of 0.74 and RMSE of 8.14 t·ha^−1^).

### 3.3. Interspecies AGB: SGB and RF Regression Predictive Performance Based all Variables

**Figure 4.**
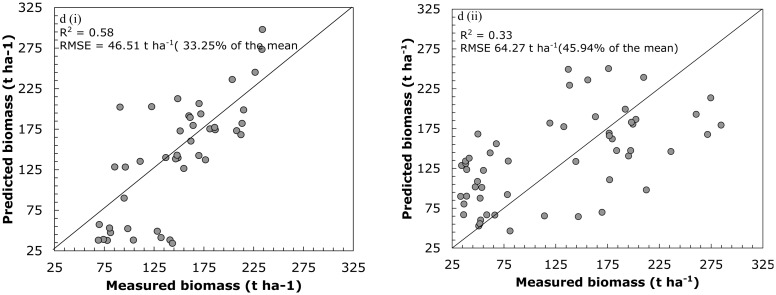
The one-to-one relationship between measured and predicted inter-species biomass for all species data combined, based on (i) SGB and (ii) RF algorithms without variable selection.

In testing the potential of SGB and RF in predicting interspecies biomass it can be observed that SGB produced plausible results based on the R^2^ of 0.58 and RMSE of 46.51 t·ha^−1^; 33.25% of the mean compared to RF which had an R^2^ of 0.33 and RMSE of 64.27 t·ha^−1^; 45.94% of the mean (Figure 4).

### 3.4. Variable Selection Using SGB and RF Models

The SGB and RF variable importance measures were used to explore the relevance of model input variables (strategically positioned RapidEye spectral bands as well as derived vegetation indices). The backward variable selection provided by the two algorithms (SGB and RF) have successfully explored and defined the relative importance of the individual input variables (predictors). Additionally, the methods further managed to select the optimal number of the input variables for predicting intra-and-inter species AGB. For SGB better results were achieved after variable selection was implemented, see Table 4. SGB backward variable selection method selected a few optimal number of important variables for (a) *E. grandis* (*p* = 4); (b) *E. dunii* (*p* = 7); (c) *P. taeda* (*p* = 6) and (d) all species data combined (*p* = 19), using the optimal *nt* and *lr* which resulted in deviance reduction (Table 3). For instance, *E. grandis* achieved the lowest predictive deviance (deviance = 0.27) based on *nt* = 2350, *lr* = 0.001 and *tc* = 3. *E. dunii* on the other hand, yielded better results (lowest deviance value) based on a value of *lr* = 0·001, *nt* = 3750 and *tc*= 3. Similarly, for *P. taeda* and all species combined, a value of *nt* = 2850, *lr* = 0.001 and *tc* = 3 produced the best results with the lowest deviance.

**Figure 5.**
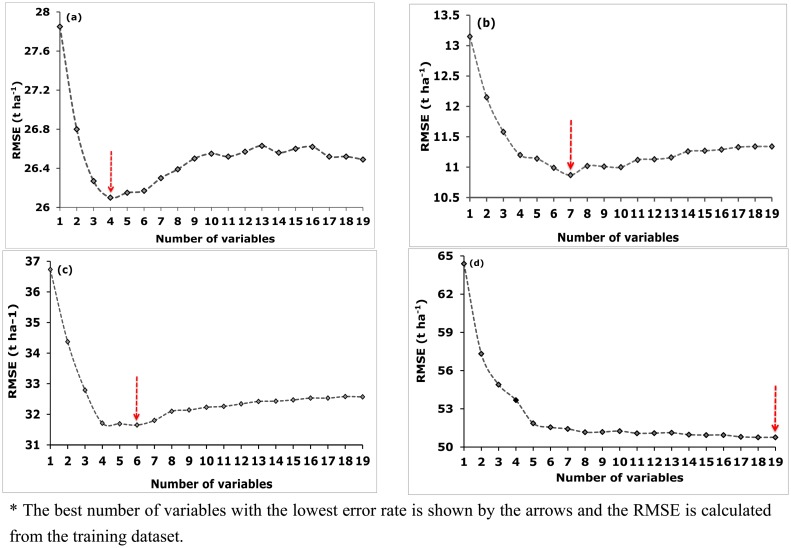
Show the optimal number of variables (spectral bands and VIs) based on the backward feature elimination search function for estimating intra-and-inter species using Random Forest (based on 1000 repetitions). In Figure 5, (**a**–**d**) represent *E. grandis, E. dunii, P. taeda* and inter-species dataset. * The best number of variables with the lowest error rate is shown by the arrows and the RMSE is calculated from the training dataset.

For the RF ensemble, the optimal number of variables was determined based on the lowest averaged RMSE obtained after running the backward feature elimination process a 1000 times. The process selected four variables for predicting (a) *E. grandis* based on averaged RMSE of 26.10 t·ha^−1^; seven predictor variables for (b) *E. dunii* based on an averaged RMSE value of 10.87 t·ha^−1^; six variables for (c) *P. taeda* with an averaged RMSE value of 31.65 t·ha^−1^ and lastly, nineteen variables for (d) the all species dataset based on a RMSE value of 50.76 t·ha^−1^ (Figure 5). The findings in Figure 5 further demonstrate that the RMSE error generally decreased as the least important variables were removed from the model progressively. The use of the most important RF selected variables produced the lowest RMSE across all species categories. To conclude, important variables selected by SGB and RF (Table 3) were used in the final model for predicting biomass across all species categories using the test dataset (Table 4).

The results in Table 3 show the most important predictor variables that were selected for estimating intra-and-inter species biomass. Most interestingly, the results from both models shows that a limited and similar number of input variables contribute to intra-and-inter species biomass prediction. It can be observed that in predicting intra-and-inter species biomass, the NIR, red-edge, and Red bands are selected across all categories by both algorithms (Table 3).

### 3.5. Intra-Species AGB: SGB and RF Regression Predictive Performance Using Selected Variables

Table 4 demonstrates inter-and-intra species aboveground biomass prediction results obtained using the SGB and RF algorithms and the most important variables shown in Table 3. It can be observed that inter-and-intra species biomass predictions based on the most important variables provides better predictive accuracies when compared to the SGB and RF models that use all the predictor variables (Figures 3 and 4) The SGB model produced good accuracies in predicting *E. grandis* (R^2^ = 0.80, RMSE = 16.93), *P. taeda* (R^2^ = 0.79, RMSE = 17.27 t·ha^−1^) and the all species data (R^2^ = 0.61, RMSE = 43.39 t·ha^−1^). The RF ensemble however, demonstrated better results (R^2^ = 0.79; RMSE 7.18 t·ha^−1^) in predicting *the biomass E. dunii* (Table 4).

## 4. Discussion

The accurate, reliable and timely quantification of intra-and-inter species AGB using remote sensing technologies is critical for better understanding the role of forests in local climate systems through biosphere-atmospheric interactions for a detailed evaluation of commercial forest resources, as well as for informed sustainable management. In this study we assessed two machine learning regression algorithms namely, SGB and RF based on 1000 model runs in predicting intra-and-inter species biomass in a commercial plantation forest located in the midlands region of KwaZulu- Natal, South Africa using the RapidEye sensor.

### 4.1. RapidEye Image Potential in Predicting Intra-and-Inter Species Biomass

One of the most critical challenges in predicting biomass in plantation forests using remote sensing is the complexity of species structural and taxonomic composition as well as the presence of dense vegetation canopies resulting in significant inter-species biomass variations. It is therefore critical to identify remote sensing datasets with critical spectral information that can overcome the saturation problems and produce better intra-and-inter species biomass prediction accuracies. In this study, we have shown that high spatial resolution RapidEye image data with strategically positioned bands can accurately predict intra-and-inter species biomass in commercial forests when compared to the existing broadband multispectral data, which have high spectral variation and saturation problems at high density biomass. Furthermore, this study demonstrated new generation multispectral sensors as having the capability to provide a better and cost-effective alternative for predicting interspecies biomass, when compared to existing broadband multispectral images [12,13,32]. Most importantly, the presence of the red-edge band, which has been unavailable in existing multispectral sensors provide very critical and sensitive measurements of vegetation properties such as chlorophyll content, necessary for predicting forest metrics, such as biomass *etc.* [13,34]. The findings from this study therefore largely supports the claim that strategically positioned bands (e.g., red-edge) found in new generation RapidEye multispectral imagery, contains more spectral information critical for vegetation mapping, when compared to other broadband multispectral sensors.

### 4.2. SGB and RF Prediction Performance Using Different RapidEye Spectral Parameters

Stochastic gradient boosting has increasingly been used in ecological modelling with limited applications in remote sensing studies e.g., [44,45,47,76,86–92]. On the other hand, random forest has been applied mainly in classification e.g., [37,38,62,93–97], hence there are limited remote sensing studies that utilize SGB and RF for regression analysis e.g., [13,18,21]. The results of the present work have demonstrated the applicability and strength of the two algorithms (SGB and RF) for variable selection and intra-and-inter species biomass prediction using the spaceborne RapidEye imagery.

Moreover, for the two different algorithms applied, the better results based on the R^2^ and RMSE were obtained from the SGB model across all species categories except for the *E. dunii* dataset. The results of the present study further demonstrated that SGB and RF models are useful and robust for intra-species biomass prediction, using remotely sensed data. For the prediction of all inter-species biomass (species data combined), the RF model performed poorly when using all the variables. We attribute this poor performance of RF to the high variability in biomass, as a result of the existing differences amongst the tree species considered in this study. The results of this study have shown that RF is less robust in environment with mixed species, when compared to the SGB algorithm. Furthermore, literature shows that the RF regression algorithm results in underestimation, when the dataset is large and variable, as well as overestimation, when the data is small with less variability [13,98].

For the SGB model algorithm, plausible interspecies biomass prediction results were observed, indicating the model's robustness in handling non-linear interspecies biomass relationships. The good performance of the SGB regression algorithm can be associated with the model's internal regularization process and the model's element of stochasticity, which is well known for enhancing the model's predictive performance [47,75,99,100]. These results are further supported by Carreiras, Vasconcelos and Lucas [21] whose work demonstrated that the simple base learner in our case, decision trees, built by running the SGB model using a random sub-sample of the training data without replacement, substantially improved the prediction accuracy. However, the effectiveness and robustness of the SGB algorithm in variable selection, based on remotely sensed data sets still needs to be tested in the mapping and understanding of other vegetation metrics such as aboveground carbon content. This information would aid in assessing forests contribution to carbon sequestration, as well as for a comprehensive evaluation of commercial forest resources, which is a pre-requisite for informed sustainable management.

## 5. Conclusion

This paper investigated: (i) the robustness of two machine learning algorithms, Stochastic Gradient Boosting and Random Forest regression trees to predict intra- and-inter species biomass in plantation forests using RapidEye multispectral imagery in KwaZulu Natal, South Africa and (ii) the performance and the strength of the SGB and RF regression algorithms as variable selection and prediction methods.

Our results have demonstrated that:

(1) Stochastic Gradient Boosting regression tree is more robust in predicting both intra-and-inter species biomass in plantation forests when integrated with the strategically positioned bands of the multispectral spaceborne RapidEye imagery as compared to the Random Forest ensemble.
(2) The new generation spaceborne multispectral sensors (e.g., RapidEye) with a high spatial resolution have the potential to satisfactorily predict intra-and-inter species biomass in areas of closed and dense vegetation.
(3) Both machine learning algorithms (SGB and RF regression trees) were able to provide a valuable screening tool for the identification of the most important spectral bands and derived vegetation indices, required accurate inter-and-intra species biomass prediction.

Overall, results of the present study demonstrate the utility, great potential and robustness of the Stochastic Gradient Boosting regression algorithm in modelling non-linear biomass relationships for mixed forests mainly based on the strategically positioned spectral information derived from the new generation multispectral sensors, a previously challenging task with broadband satellite sensors. However, there is need to further test the performance and robustness of this method (*i.e.*, SGB regression algorithm) in mapping and understanding the spatial distribution of critical forest parameters such as aboveground carbon content.

## Figures and Tables

**Table 1.** Selected strategically positioned Rapideye spectral parameters and vegetation indices used for this study.

**Parameters**	**Formula**	**References**
**Single band reflectance**		
Blue, green, red, NIR and Red-edge	-	

**Vegetation Indices**		
Simple Ratio	NIR/Red	Jordan [64]
RVI.RE (Ratio Vegetation Index)	Red-edge/NIR	de Sousa, *et al.* [65]
NDVI (Normalized Difference Vegetation Index)	(NIR−Red)/(NIR + Red)	Rouse, *et al.* [66]; Jordan [64]
NDVI.RE	(NIR − Red-edge)/(NIR + Red-edge)	Mutanga, Adam and Cho [13]-
DVI (Difference Vegetation Index)	NIR − Red	Tucker [67]
MSR (Modified Simple Ratio)	(NIR/Red)-1/(NIR/Red)^∧^°^.5^ + 1	Qi, *et al.* [68]
MSR.RE	(NIR/Red-edge)-1/(NIR /Red-edge)^∧^°^.5^ + 1	
TVI (Triangular Vegetation Index)	0.5*[120*(NIR − Green)−200*(Red-Green)]	Broge and Leblanc [69]
TVI.RE	0.5*[120*(NIR − Green)−200*(Red-edge-Green)]	
IPVI (Perpendicular Vegetation Index)	NIR/(NIR + Red)	Crippen [70]
IPVI.RE	NIR/(NIR + Red-edge)	
GI (Greenness Index)	Green/Red	Zarco-Tejada, *et al.* [71]
GI.RE	Green/Red-edge	
PSSR (Pigment specific simple ratio)	NIR/Red-edge	Blackburn [72]

**Table 2.** Descriptive statistics of the measured above ground biomass (t·ha^−1^).

**Species Type**	**Total**	**Min.**	**Max.**	**Mean**	**Std dev.**
*E. dunii*	63	33.24	96.49	52.86	16.39
*E. grandis*	65	106.03	225.07	170.30	29.94
*P. taeda*	53	137.11	298.04	206.07	42.83
*All species*	181	33.24	298.04	139.89	72.22

**Table 3.** Illustrates the most important variables retained by SGB and RF after implementing variable selection.

**Variables Selected**	***E. grandis***		***E. dunii***		***P. taeda***		***All species***	
			
	**SGB**	**RF**	**SGB**	**RF**	**SGB**	**RF**	**SGB**	**RF**
1	RE	NIR	NIR	RE	NIR	NIR	All variables selected	
2	PSSR	RE	RE	PSSR	Green	Green		
3	GI.RE	PSSR	Red	GI.RE	RE	RE		
4	NIR	DVI	GI.RE	NIR	Red	Red		
5	Green	-	Green	Green	DVI	DVI		
6	-	-	Blue	DVI		Blue		
7	-	-	-	Blue	-	-		

**Table 4.** Inter-and-intra species biomass prediction results using the most important variables selected by the two regression models SGB and RF.

**Species**	**Statistical Methods**	**tc**	**lr**	**mtry**	**nt/ntree**	**R^2^**	**RMSE (t•ha^−1^)**
*E. grandis*	SGB (n = 5)	3	0.001	-	3750	0.80	16.93
	RF (n = 4)	-	-	4	500	0.76	18.61
*E.dunii*	SGB (n = 6)	5	0.001	-	2350	0.88	09.23
	RF (n = 7)	-	-	7	500	0.79	07.18
*P.taeda*	SGB (n = 5)	5	0.001	-	800	0.79	17.27
	RF (n = 6)	-	-	6	2800	0.80	22.43
All species	SGB (n = 19)	5	0.001	-	2800	0.61	43.39
	RF (n = 19)	-	-	19	750	0.37	59.27
